# Adherence to pro-vegetarian dietary patterns and gastric cancer risk: a pooled analysis of the StoP Consortium

**DOI:** 10.1007/s10120-026-01757-4

**Published:** 2026-05-14

**Authors:** Alejandro Oncina-Cánovas, Laura Torres-Collado, Manuela García-de-la-Hera, Sandra González-Palacios, Carolina Ojeda-Belokon, Eva Negri, Claudio Pelucchi, Areti Lagiou, Pagona Lagiou, Nuno Lunet, Samantha Morais, Vicente Martin, Gemma Castaño-Vinyals, Lizbeth López-Carrillo, Raúl Ulises Hernández-Ramírez, M. Constanza Camargo, Maria Paula Curado, Zuo-Feng Zhang, Carlo La Vecchia, Paolo Boffetta, Jesús Vioque

**Affiliations:** 1 Nutritional Epidemiology Unit, Dpto. Salud Pública, Hª de La Ciencia y Ginecología, Facultad de Medicina, Universidad Miguel Hernández (UMH), Avda. Ramón y Cajal S/N, 03550 Sant Joan d’Alacant, Alicante, Spain; 2 Instituto de Investigación Sanitaria y Biomédica de Alicante (ISABIAL), Alicante, Spain; 3 Consortium for Biomedical Research in Epidemiology and Public Health (CIBERESP), Madrid, Spain; 4 Department of Medical and Surgical Sciences, University of Bologna, Bologna, Italy; 5 Department of Clinical Sciences and Community Health, University of Milan, Milan, Italy; 6 Department of Public and Community Health, School of Public Health, University of West Attica, Athens, Greece; 7 Department of Hygiene, Epidemiology and Medical Statistics, School of Medicine, National and Kapodistrian University of Athens, Athens, Greece; 8 EPIUnit ITR, Instituto de Saúde Pública da Universidade do Porto, Universidade do Porto, Porto, Portugal; 9 Departamento de Ciências da Saúde Pública e Forenses e Educaçäo Médica, Faculdade de Medicina da Universidade do Porto, Porto, Portugal; 10 Group of Investigation in Interactions Gene-Environment and Health (GIIGAS), Institute of Biomedicine (IBIOMED), Universidad de León, León, Spain; 11 Barcelona Institute for Global Health—ISGlobal, Barcelona, Spain; 12 Universitat Pompeu Fabra (UPF), Barcelona, Spain; 13 Population Health Research Center, Mexico National Institute of Public Health, Morelos, Mexico; 14 Department of Biostatistics, Yale School of Public Health, Yale School of Medicine, New Haven, CT USA; 15 Division of Cancer Epidemiology and Genetics, National Cancer Institute, National Institutes of Health, Rockville, MD USA; 16 Centro Internacional de Pesquisa, A. C. Camargo Cancer Center, São Paulo, Brazil; 17 Department of Epidemiology, UCLA Fielding School of Public Health and Jonsson Comprehensive Cancer Center, Los Angeles, CA USA; 18 Stony Brook Cancer Center, Stony Brook University, Stony Brook, NY USA

**Keywords:** Diet, Plant-based, Dietary patterns, Stomach neoplasms

## Abstract

**Background:**

Pro-vegetarian (PVG) diets may reduce gastric cancer (GC) risk, but evidence remains limited. We aimed to evaluate the association between three predefined PVG patterns—general (gPVG), healthful (hPVG), and unhealthful (uPVG)—and GC risk stratifying by sex in the context of the Stomach Cancer Pooling (StoP) Project Consortium.

**Methods:**

We analysed data from six case–control studies from the StoP Consortium. The final sample included 1,857 incidents, histologically confirmed GC cases and 5,646 controls. Food intakes were assessed using country-specific food frequency questionnaires, which allowed the estimation of PVG patterns using established scoring methods. Adherence to PVG dietary patterns was classified into quintiles. Logistic mixed models with random intercepts for each study were used to estimate odds ratios (ORs) and 95% confidence intervals (CIs).

**Results:**

Compared to the lowest quintile, the highest quintile of adherence to gPVG was associated with an OR_Q5vsQ1_ = 0.61 (95% CI: 0.50–0.75; p-trend =  < 0.001) and the hPVG was associated with an OR_Q5vsQ1_ = 0.66 (0.54–0.80; p-trend =  < 0.001). The uPVG pattern was associated with an OR_Q5vsQ1_ = 1.27 (1.05–1.55; p-trend = 0.013). The inverse association for gPVG was more evident in men, OR_Q5vsQ1_ = 0.56 (0.43–0.73) than in women, OR_Q5vsQ1_ = 0.73 (0.53–1.01); for the hPVG pattern, a significant inverse association was only observed in women, OR_Q5vsQ1_ = 0.50 (0.36–0.69); and, for the uPVG pattern, the increased GC risk was only observed in women, OR_Q5vsQ1_ = 1.70 (1.25–2.32).

**Conclusions:**

Higher adherence to gPVG and hPVG dietary patterns is associated with lower GC risk, whereas higher adherence to uPVG is associated with increased risk. The inverse association observed for hPVG pattern and the positive association for uPVG were only observed in women. These differential effects of dietary patterns in men and women deserve to be further investigated.

**Supplementary Information:**

The online version contains supplementary material available at 10.1007/s10120-026-01757-4.

## Introduction

Digestive cancers are of particular interest in public health due to their high mortality rates, particularly in men [1] and gastric cancer (GC) stands out as one of the most lethal of these cancers [2]. Various factors have been associated with the development of GC, including *Helicobacter pylori* infection [3], smoking [4, 5], genetic predisposition [6], socioeconomic status [7] and dietary habits [8]. Regarding diet, salty foods and high alcohol consumption have been linked to an increased risk [9, 10], whereas intake of fruit and vegetables [11], and green tea [12], have been inversely associated with GC risk. However, diet has not been sufficiently explored as a whole, and the impact of specific dietary patterns on the risk of GC is not yet fully understood.

Plant-based dietary patterns have become common in dietary guidelines worldwide [13]. Vegetarian diets, in particular, have gained popularity over the last decade, not only due to their environmental and ethical implications [14], but also because of their potential health benefits [15]. A recent analysis involving more than 70,000 participants from the Adventist Health Study found that vegetarians had a 45% lower risk of GC compared with nonvegetarians [16]. Despite this, adherence to vegetarian diets remains low, prompting the development of more flexible pro-vegetarian (PVG) dietary patterns that prioritize plant-based foods without strictly excluding animal products [17].

Healthful (hPVG) and unhealthful (uPVG) variations of PVG dietary patterns have also been developed [18]. These patterns have been associated with some digestive cancers [19–21], such as colorectal cancer [22], but their contribution to other types, like GC, remains understudied. A large-scale cohort study conducted within the UK Biobank, with over 100,000 participants, examined the association between hPVG, among other healthy dietary patterns, and the incidence of gastrointestinal cancers, including GC. The results showed that a higher adherence to an hPVG pattern was associated with 49% lower risk of GC [23]. Another study in a Spanish population participating in the PANESOES project, also examined the association between general PVG (gPVG), hPVG and uPVG patterns and oesophageal, gastric and pancreatic cancers. A protective association was observed for the gPVG and hPVG patterns with GC, whereas the uPVG version was associated with a higher risk of GC [24]. Despite this, these associations differ by sex remains largely unexplored.

Therefore, it is necessary to expand the study of this association using a larger sample, such as that included in the Stomach Cancer Pooling (StoP) Project Consortium. This is an international consortium of epidemiological studies on GC [25, 26], which offers a unique opportunity to investigate the role of dietary patterns, including the PVG dietary patterns, by pooling data from several studies to obtain a large sample size and a better control of confounding. Thus, the aim of our study was to evaluate the association between three predefined PVG dietary patterns (gPVG, hPVG and uPVG) and the risk of GC differentiating by sex.

## Methods

### Study design and population

The present analysis used information from studies included in the StoP Project dataset (http://www.stop-project.org/), an international consortium established in 2012 to investigate lifestyle, dietary, and genetic determinants of GC. The StoP Consortium includes 34 case–control or nested case–control studies from 15 countries, providing a total of 13,121 histologically confirmed GC cases and 31,420 controls. Original study databases, questionnaires, and related information were transferred to the Coordinating Centre in Milan under signed data-sharing agreements, and all variables were harmonized according to a pre-specified format to ensure consistency and comparability. Ethical approval for the project was granted by the University of Milan Institutional Review Board (reference no. 19/15, dated 01/04/2015), and all individual studies were conducted in accordance with the Declaration of Helsinki and applicable regulations on the protection of human subjects.

In our study, we included a total of 1,857 cases of GC (1,167 men and 690 women) and 5,646 controls (3,034 men and 2,612 women), from 6 studies with the dietary data needed to create PVG dietary patterns: one each from Italy [27], Greece [28], Portugal [29] and Mexico [30], and two from Spain [31, 32]. Of these, three used hospital-based controls and the other three used population-based controls. More details about the included studies are shown in Table 1.

**Table 1:** Main characteristics of the studies included in our analysis from the StoP-Consortium

Study country	Control type	Period of recruitment	Gastric cancer cases	Controls	FFQ nº items
Study 1_Italy [27]	Hospital-based	1997–2007	218	526	78
Study 2_Greece [28]	Hospital-based	1981–1984	106	99	80
Study 3_Portugal [29]	Population-based	1999–2006	591	1436	82
Study 4_Spain_1 [31]	Population-based	2008–2012	396	452	140
Study 5_Spain_2 [32]	Hospital-based	1995–1999	234	454	93
Study 6_Mexico [30]	Population-based	2004–2005	312	2679	127

FFQ, Food Frequency Questionnaire

### Pro-vegetarian dietary patterns

The dietary intake of each participant was assessed using country-specific food frequency questionnaires (FFQs). Each FFQ included standard portion sizes and a list of the most commonly consumed foods in the region. The number of items in each FFQ differed across studies (Table 1), although all FFQs comprised information on the key food groups required to construct the PVG patterns. To estimate intake in grams for each food item, two dietitians independently harmonized consumption frequencies for each study. Foods reported as mixed dishes (e.g., “Pasta with ragù sauce” in the Italian study or “A taco al pastor” in the Mexican study) were not classified into any group because their ingredients could fit into multiple food groups. Once gram intakes were estimated, individual foods were classified into the main food groups considered in the PVG patterns through consensus among three dietitians. After that, different PVG patterns were created following the methodology of previous authors: For the gPVG pattern, we applied the method proposed by Martínez-Gonzalez et al. [17] and for the two derived patterns, hPVG and uPVG, we followed the approach described by Satija et al. [18]. For hPVG and uPVG three modifications were made: (1) the “Vegetable oils” food group was replaced by “Olive oil”, as olive oil was the predominant culinary fat in the participating countries; (2) the “Potatoes” food group was divided according to cooking method into two food groups “Fries and chips” and “Boiled/Baked potatoes”, acknowledging that fried potatoes have been consistently associated with adverse health outcomes, whereas the evidence regarding boiled/baked potatoes remains mixed; (3) and the group “Miscellaneous animal-based food group” was excluded due to its heterogeneity and difficulty of classification as plant- or animal-based.

To construct the PVG dietary patterns, once food consumption data in grams were obtained for each participant in their respective study, we classified the food items into 18 groups, of which 13 were plant-based and 5 were animal-based. When information on a food group was not available in a given study, an intake value of 0 was assigned. Details regarding the food items included in each food group and the scoring criteria of each PVG pattern by study can be found in Supplementary Tables 1–6.

Subsequently, consumption of each food group (in grams) was energy-adjusted for each study using the residual method [33]. Calorie-adjusted intakes were then categorized into quintiles and assigned scores from 1 to 5, according to level of consumption. The gPVG pattern only considered whether foods were plant- or animal-based. It included 7 plant components (vegetables; fruits; legumes; cereals, both refined and whole; potatoes, both boiled/baked and fried; nuts; olive oil) and 5 animal components (meat and meat products; animal fats; eggs; fish and seafood; dairy products). Plant foods scored positively, with higher consumption corresponding to higher scores (the maximum being 5 points). Animal foods scored inversely, with higher consumption corresponding to lower scores (the minimum being 1 point). The hPVG and uPVG patterns also considered the healthfulness of plant-based foods, based on their association with obesity, diabetes, cardiovascular disease, and/or cancer [18]. The hPVG pattern scored healthy plant foods positively and unhealthy plant foods negatively, while the uPVG pattern scored inversely: unhealthy plant foods positively and healthy plant foods negatively. For these patterns, cereals were divided into whole grains (healthy) and refined grains (unhealthy), while potatoes were divided into boiled/baked (healthy) and fried/chips (unhealthy). Both hPVG and uPVG included four additional food groups: tea and coffee (healthy), fruit juices (unhealthy), sugar-sweetened beverages (unhealthy), and desserts and sweets (unhealthy). To obtain the final scores of the gPVG and hPVG/uPVG patterns we summed the values of 12 and 18 food groups, respectively. This resulted in continuous adherence scores ranging from 12–60 points (gPVG) and 18–90 points (hPVG and uPVG) for each participant. The quintile ranges for each study population can be found in Supplementary Table 7.

Finally, to analyse the pooled association of PVG adherence across all participants, we combined scores from each study in a single database and classified adherence in quintiles. Participants reporting implausible energy intakes (< 800 or > 4000 kcal/day for men; < 500 or > 3500 kcal/day for women) [34] were excluded.

### Covariates

Sociodemographic and lifestyle variables were collected and harmonized using a standardized format: age (< 49; 50–59; 60–69; ≥ 70 years), sex (men; women), social class based on educational level (low, less than secondary; intermediate, secondary; high, more than secondary), alcohol intake (0; 1–2; ≥ 3 drinks/day), smoking status (never; former; current), *Helicobacter pylori* infection (seronegative, seropositive, missing data) and total energy intake (kcal/day). In addition, for GC cases, information on histological type (diffuse; intestinal; other) and anatomical location (cardia; non-cardia) was also collected and used.

### Statistical analysis

We described the main characteristics of the participants according to their classification as cases or controls and stratifying by sex. For continuous variables, both the mean and standard deviation (SD) were reported, while for categorical variables, the frequency (n) and percentage (%) were provided.

We conducted a one-stage pooled analysis to examine the associations between the three PVG patterns (gPVG, hPVG, and uPVG) and GC risk and explored this association by sex. Logistic mixed-effects models with random intercepts for study were used to estimate odds ratios (ORs) and 95% confidence intervals (CIs) of GC across quintiles of PVG adherence. The ORs and the corresponding 95% CIs were also estimated by considering quintiles as an ordinal variable (per quintile increment), and per each 5-point increase in adherence. The first quintile (Q1) was the reference category in all analyses. We reported only a multivariable adjusted model. This model included sex (only for total sample analysis) and age, which were common matching variables in all the included studies, plus social class based on educational level, smoking status, alcohol consumption and *Helicobacter pylori* serostatus (treated as a three-category variable in the analyses: seronegative / seropositive / missing data), all of which are well-known risk factors for GC in the scientific literature, and energy intake.

In addition, we performed stratified analyses to explore the effect of adherence to the three PVG dietary patterns by histological subtype (intestinal, diffuse, other types) and anatomical subsite (cardia, non-cardia). Sensitivity analyses were also performed to assess the robustness of the association between adherence to PVG dietary patterns and GC risk. First, we sequentially excluded each study from the StoP Consortium. Second, we stratified the analysis by type of control (hospital-based or population-based). Third, we repeated the main analysis excluding participants with missing data for *Helicobacter pylori* infection status. Finally, we conducted a stratified analysis according to *Helicobacter pylori* infection status.

All analyses were conducted using STATA software, version 17.

## Results

The main sociodemographic and lifestyle characteristics of the 1,857 GC cases and 5,646 controls included in the analysis are shown in Table 2, overall and stratified by sex. A higher proportion of cases (34.1%) were ≥ 70 years compared to controls (28.8%), with women showing the largest age gap between cases and controls (35.4% vs 26.2%). Cases were predominantly men (62.8%), whereas there was a more balanced sex distribution among controls (53.7% men). With regard to social class, the imbalance between cases and controls was more pronounced in women (73.3% vs 52.5% in the lower class) than in men (67.0% vs 48.9%). Alcohol consumption differed markedly by sex: whereas ≥ 3 drinks/day was common among men cases (53.5%) and controls (39.4%), it was substantially lower in women (10.3% and 6.1%, respectively). Similarly, tobacco smoking showed a clear sex pattern, with current smoking more prevalent in men (31.9% of men cases vs 9.3% of women cases). Cases had a lower seropositivity for Helicobacter pylori than controls in both sexes, with no notable sex differences. Mean energy intake was consistently higher in men than women, both among cases (2,293.1 vs 1,955.6 kcal/day) and controls (2,155.8 vs 1,853.1 kcal/day). Regarding PVG dietary scores, cases showed lower gPVG and hPVG scores than controls across both sexes, though the difference in hPVG was more notable in women (53.2 vs 55.0) than in men (53.7 vs 53.4). The uPVG score was higher among women cases compared to women controls (54.2 vs 52.5), whereas men cases and controls showed similar scores (55.7 vs 55.6).

**Table 2:** Sociodemographic characteristics, lifestyle habits and pro-vegetarian dietary patterns among controls and gastric cancer cases of the StoP Consortium (*n* = 7,503)

Variables	Total sample		Men		Women	
	Controls (*n* = 5,646)	Cases (*n* = 1,857)	Controls (*n* = 3,034)	Cases (*n* = 1,167)	Controls (*n* = 2,612)	Cases (*n* = 690)
Age (in years), *n* (%)						
< 49	1083 (19.2)	304 (16.4)	444 (14.6)	170 (14.6)	639 (24.5)	134 (19.4)
50—59	1155 (20.4)	377 (20.3)	587 (19.4)	253 (21.7)	568(21.7)	124 (18.0)
60–69	1784 (31.6)	543 (29.2)	1062 (35.0)	355 (30.4)	722 (27.6)	188 (27.2)
≥ 70	1624 (28.8)	633 (34.1)	941 (31.0)	389 (33.3)	683 (26.2)	244 (35.4)
Social class, *n* (%)						
Low	2852 (50.5)	1288 (69.4)	1482 (48.9)	782 (67.0)	1,370 (52.5)	506 (73.3)
Intermediate	1454 (25.8)	361 (19.4)	790 (26.0)	247 (21.2)	664 (25.4)	114 (16.5)
High	1340 (23.7)	208 (11.2)	762 (25.1)	138 (11.8)	578 (22.1)	70 (10.2)
Alcohol consumption (drinks/day), *n* (%)						
0	1376 (24.4)	443 (23.9)	370 (12.2)	125 (10.7)	1,006 (38.5)	318 (46.1)
1–2	2915 (51.6)	719 (38.7)	1469 (48.4)	418 (35.8)	1446 (55.4)	301 (43.6)
≥ 3	1355 (24.0)	695 (37.4)	1195 (39.4)	624 (53.5)	160 (6.1)	71 (10.3)
Tobacco smoking, *n* (%)						
Never	2716 (48.1)	889 (47.9)	862 (28.4)	320 (27.4)	1854 (71.0)	569 (82.5)
Former	1727 (30.6)	532 (28.6)	1374 (45.3)	475 (40.7)	353 (13.5)	57 (8.2)
Current	1203 (21.3)	436 (23.5)	798 (26.3)	372 (31.9)	405 (15.5)	64 (9.3)
*Helicobacter pylori*, *n* (%)						
Seronegative	442 (7.8)	153 (8.2)	191 (6.3)	84 (7.2)	251 (9.6)	69 (10.0)
Seropositive	2869 (50.8)	687 (37.0)	1546 (51.0)	433 (37.1)	1323 (50.7)	254 (36.8)
Missing data	2335 (41.4)	1017 (54.8)	1297 (42.7)	650 (55.7)	1038 (39.7)	367 (53.2)
Energy intake, kcals/day^a^	2015.7 (586.4)	2167.7 (629.6)	2155.8 (606.9)	2293.1 (625.8)	1853.1 (516.1)	1955.6 (577.3)
Points of score^a^						
gPVG	41.3 (5.7)	40.1 (5.9)	41.0 (5.7)	40.1 (5.8)	41.6 (5.7)	40.0 (6.0)
hPVG	54.1 (7.1)	53.5 (6.7)	53.4 (7.1)	53.7 (6.7)	55.0 (7.1)	53.2 (6.8)
uPVG	54.2 (7.2)	55.2 (6.9)	55.6 (7.0)	55.7 (6.7)	52.5 (7.2)	54.2 (7.1)

SD, Standard Deviation; c/day, cigarettes per day; gPVG, general pro–vegetarian dietary pattern; hPVG, healthful pro–vegetarian dietary pattern; uPVG, unhealthful pro–vegetarian dietary pattern

^a^Mean (SD) (all such values)

We found an inverse association between adherence to the gPVG and hPVG dietary patterns and GC, while higher adherence to uPVG was associated with a higher risk (Table 3). After adjusting for age, social class, smoking status, alcohol consumption, energy intake and *Helicobacter pylori* serostatus, participants in the highest quintile of gPVG adherence had an OR_Q5vsQ1_ of 0.61 (95% CI: 0.50–0.75; p-trend < 0.001). The highest adherence to the hPVG pattern was also associated with lower GC risk, OR_Q5vsQ1_ = 0.66 (95% CI: 0.54–0.80; p-trend < 0.001). Conversely, the highest quintile of adherence to the uPVG pattern was associated with a higher risk, OR_Q5vsQ1_ = 1.27 (95% CI: 1.05–1.55; p-trend = 0.013). Per 1-quintile increment in adherence, the ORs of GC were 0.89 (95% CI: 0.85–0.93) for gPVG, 0.91 (95% CI: 0.87–0.95) for hPVG and 1.06 (95% CI: 1.01–1.10) for uPVG. Per 5-point increment in adherence, results were consistent, with corresponding ORs of 0.86 (95% CI: 0.81–0.91), 0.89 (95% CI: 0.85–0.93) and 1.05 (95% CI: 1.01–1.10), respectively.

**Table 3:** Odds ratios and 95% confidence intervals of gastric cancer according to the adherence to pro-vegetarian dietary patterns (in quintiles, Q) stratified by sex^a^ in the StoP Consortium

	gPVG dietary pattern						
	Q1 (< 37)^b^	Q2 (37–40)	Q3 (41–43)	Q4 (44–46)	Q5 (≥ 47)	Per 1 quintile increment	Per 5 points increment in adherence
Controls/cases	1,152/505	1,329/456	1,171/377	950/259	1,044/260		
Total sample	1 (Ref.)	0.81 (0.68–0.95)	0.78 (0.65–0.94)	0.68 (0.56–0.83)	0.61 (0.50–0.75)	0.89 (0.85–0.93)	0.86 (0.81–0.91)
Controls/cases	643/309	741/296	443/167	682/238	525/157		
Only men	1 (Ref.)	0.78 (0.63–0.96)	0.74 (0.58–0.95)	0.70 (0.55–0.88)	0.56 (0.43–0.73)	0.88 (0.84–0.94)	0.86 (0.80–0.92)
Controls/cases	509/196	588/160	353/97	643/134	519/103		
Only women	1 (Ref.)	0.88 (0.67–1.16)	0.96 (0.70–1.33)	0.75 (0.56–1.01)	0.73 (0.53–1.01)	0.92 (0.86–0.99)	0.88 (0.80–0.96)

	Q1 (< 49)^b^	Q2 (49–52)	Q3 (53–56)	Q4 (57–60)	Q5 (≥ 61)	Per 1 quintile increment	Per 5 points increment in adherence
hPVG dietary pattern							
Controls/cases	1,255/420	1,085/399	1,262/433	991/322	1,053/283		
Total sample	1 (Ref.)	0.91 (0.77–1.08)	0.85 (0.72–1.01)	0.80 (0.66–0.96)	0.66 (0.54–0.80)	0.91 (0.87–0.95)	0.89 (0.85–0.93)
Controls/cases	756/248	613/259	678/273	502/193	485/194		
Only men	1 (Ref.)	1.01 (0.81–1.26)	0.95 (0.76–1.19)	0.85 (0.67–1.08)	0.83 (0.64–1.06)	0.95 (0.89–1.00)	0.93 (0.88–0.98)
Controls/cases	499/172	472/140	584/160	489/129	568/89		
Only women	1 (Ref.)	0.80 (0.60–1.05)	0.74 (0.56–97)	0.75 (0.56–1.00)	0.50 (0.36–0.69)	0.87 (0.81–0.93)	0.84 (0.79–0.91)

	Q1 (< 49)^b^	Q2 (49–53)	Q3 (54–56)	Q4 (57–60)	Q5 (≥ 61)	Per 1 quintile increment	Per 5 points increment in adherence
uPVG dietary pattern							
Controls/cases	1,254/307	1,348/431	926/334	1,028/377	1,090/408		
Total sample	1 (Ref.)	1.13 (0.94–1.35)	1.22 (1.01–1.48)	1.22 (1.01–1.48)	1.27 (1.05–1.55)	1.06 (1.01–1.10)	1.05 (1.01–1.10)
Controls/cases	474/162	700/263	497/225	624/241	739/276		
Only men	1 (Ref.)	1.01 (0.78–1.28)	1.17 (0.90–1.52)	1.02 (0.79–1.32)	1.02 (0.79–1.32)	1.00 (0.95–1.06)	1.00 (0.94–1.06)
Controls/cases	780/145	648/168	429/109	404/136	351/132		
Only women	1 (Ref.)	1.23 (0.94–1.61)	1.18 (0.87–1.59)	1.48 (1.10–1.98)	1.70 (1.25–2.32)	1.13 (1.05–1.21)	1.13 (1.05–1.21)

Model adjusted by age (< 49; 50–59; 60–69; ≥ 70 years), social class (low; intermediate; high), tobacco smoking (never; former; current), alcohol consumption (0; 1–2, ≥ 3 drinks/day), energy intake (kcal/day) and *Helicobacter pylori* infection (seronegative, seropositive, missing). For whole sample, the adjusted models included sex as a covariate.

gPVG, general pro–vegetarian dietary pattern; hPVG, healthful pro–vegetarian dietary pattern; uPVG, unhealthful pro–vegetarian dietary pattern

^a^Men, *n* = 4,201; women, *n* = 3302

^b^Adherence range points

When analyses were stratified by sex (Table 3), stronger inverse association with gPVG pattern was observed in men than in women, OR_Q5vsQ1_ = 0.56 (95% CI: 0.43–0.73) vs OR_Q5vsQ1_ = 0.73 (95% CI: 0.53–1.01). For the hPVG pattern, the inverse association was more pronounced in women, OR_Q5vsQ1_ = 0.50 (95% CI: 0.36–0.69), than in men, OR_Q5vsQ1_ = 0.83 (95% CI: 0.64–1.06). For the uPVG pattern, an increased GC risk was observed in women, OR_Q5vsQ1_ = 1.70 (95% CI: 1.25–2.32), but not in men, OR_Q5vsQ1_ = 1.02 (95% CI: 0.79–1.32).

We also evaluated the association between food groups included in PVG dietary patterns and GC risk (Supplementary Table 8). A higher consumption of vegetables, fruits, tea and coffee, and olive oil was associated with a lower risk, whereas higher intake of meat, meat products and refined grains was associated with an increased risk. Potato consumption showed a different association depending on preparation method, with boiled potatoes positively associated with GC risk and fried potatoes, inversely associated (Supplementary Fig. 1).

Supplementary Table 9 shows the associations between PVG patterns and GC risk by histological subtype. The gPVG and hPVG dietary patterns were inversely associated with the risk of intestinal- and diffuse-type GC, whereas uPVG showed a positive association with diffuse-type GC. Supplementary Table 10 shows the associations between PVG patterns and GC risk by anatomical subsite. Adherence to gPVG was inversely associated with both cardia and non-cardia GC, hPVG was inversely associated with non-cardia GC, while uPVG was positively associated with cardia GC. Similar results were observed when we evaluated the association by histological type and anatomical subsite. The hPVG pattern showed a stronger inverse association for diffuse-type non-cardia GC, whereas the uPVG pattern was positively associated mainly with diffuse-type non-cardia GC (Supplementary Table 11).

Finally, during sensitivity analyses, the association between adherence to PVG dietary patterns and GC risk remained stable after the sequential exclusion of each study (Supplementary Table 12), when we differentiated between hospital-based and population-based controls (Supplementary Table 13) and after excluding participants with missing data for *Helicobacter pylori* status (Supplementary Table 14). Stratified analysis by *Helicobacter pylori* serostatus showed similar results (Supplementary Table 15).

## Discussion

Our study including 1,857 GC cases and 5,646 controls shows an inverse association between adherence to gPVG and hPVG dietary patterns and GC risk, while a higher adherence to the uPVG was positively associated. Sex-stratified analyses revealed that the inverse association for gPVG was present in both sexes, while the protective effect of hPVG was more pronounced in women and the positive association of uPVG was driven predominantly by women. These associations were consistent across the different histological subtypes, cardia and non-cardia gastric cancer locations, and when restricting to participants with available *Helicobacter pylori* infection status data.

Our main results are consistent with most previous evidence. A study conducted in three large North American cohorts (the Nurses’ Health Study, Nurses’ Health Study II, and Health Professionals Follow-up Study) investigated the association of three plant-based dietary indices (PDIs) with the risk of several digestive cancers [19]. Each additional 10 points of adherence to the overall PDI and healthful PDI (hPDI) was associated with a 6% and 7% lower risk of all digestive cancers. However, when GC was analysed separately, no significant association with any of the PDIs was observed. An analysis conducted within a network of Italian case–control studies, including 230 GC cases (included in the StoP dataset), also evaluated the association with PDIs by tertiles of adherence showing an inverse association between the highest tertile of adherence to the hPDI and the risk of GC, OR = 0.42 (0.27–0.67). The results for the overall PDI and uPDI patterns were similar to those observed in our study [35]. A recently published study based on a Chinese cohort of over 40,000 participants [36], which included 355 GC incident-cases, examined the association between diet quality, including PDI and uPDI patterns, and upper gastrointestinal cancers, and obtained results consistent with ours. In that case, the highest quartile of adherence to the overall PDI pattern was associated with a hazard ratio (HR) of 0.49 (95% CI: 0.30–0.81), while the highest adherence to the uPDI increased the GC risk, HR = 2.28 (95% CI 1.40–3.74).

Several mechanisms can explain the protective association shown by gPVG and hPVG. First, these dietary patterns assigned a positive score to plant-based foods such as fruit, vegetables, legumes, whole-grain cereals, nuts and olive oil, all of which are important sources of nutrients and bioactive compounds with anticarcinogenic properties [37]. Second, these food-groups are also rich in fibre, which may lower the risk of GC by diluting and binding carcinogens, regulating gastric acidity, and stimulating the production of short-chain fatty acids with anti-inflammatory effects [38]. Third, antioxidants and phytochemicals abundant in plant-based foods, including carotenoids, flavonoids, and lignans, may attenuate oxidative stress and modulate key pathways involved in cell proliferation and apoptosis [39]. Fourth, healthy sources of unsaturated fatty acids, especially those from nuts and olive oil, may also contribute to reducing GC risk by reducing inflammation and strengthening the immune system [40]. Finally, these plant-based foods create a gastric environment that is less favourable for the colonization and growth of *Helicobacter pylori* by increasing the production of protective mucus, modulating chronic inflammation, and providing compounds with natural antimicrobial activity (such as certain polyphenols and sulfuric compounds from vegetables) [39]. Sex differences in the observed associations may partly reflect hormonal influences, as oestrogen has been suggested to exert gastroprotective effects through anti-inflammatory and antioxidant pathways, potentially amplifying the benefits of healthful plant-based diets in women [41].

The uPVG pattern is also a PVG pattern, although characterized by a high intake of fried potatoes, sugar-sweetened beverages, refined cereals, desserts, and sweets, all of which are linked to an increased risk of GC through several mechanisms. Several of these foods, classified in the literature as ultra-processed foods (UPF), are major sources of free sugars and saturated or trans fats, which promote insulin resistance, obesity, which is a risk factor for GC [42], and systemic inflammation [43]. Thermal processing methods used in these UPF, such as deep frying or baking at high temperatures can generate carcinogenic compounds, including advanced glycation end products, and heterocyclic amines, all of which can damage gastric mucosa and promote carcinogenesis [44]. The high levels of sugars and refined starches in UPF may also alter the gastric microenvironment, supporting the growth and virulence of *Helicobacter pylori* by increasing the availability of fermentable substrates and modifying local pH [45]. Finally, the low nutrient density and lack of protective phytochemicals in UPF may aggravate oxidative stress and weaken the body’s ability to counteract carcinogenic harm [43]. The stronger positive association of uPVG with GC in women warrants attention. Women in our sample showed considerably lower alcohol and tobacco consumption than men, suggesting that diet may represent a relatively more important determinant of GC risk in women when other major risk factors are less prevalent.

Our study has limitations. First, the country-specific FFQs differed in food lists and portion sizes, which is a source of heterogeneity in our dietary variables. Second, we assumed zero intake during the construction of PVG dietary patterns when information on a food group was not available, which could have underestimated consumption in some populations. In this sense, although quintiles of adherence to PVG patterns were derived from pooled distribution, cross-country variation in absolute intake levels should be considered when interpreting adherence scores. To facilitate interpretation, Supplementary Table 7 provides the country-specific adherence ranges calculated independently within each study. Third, information on *Helicobacter pylori* infection was based on serological markers, which do not distinguish between current and past infection and do not capture previous eradication therapy. In addition, missing data on *Helicobacter pylori* status in a high proportion of participants (44.7%) may have led to some residual confounding despite adjustment in the analyses, although we performed sensitivity analysis only with those participants with information for *Helicobacter pylori* serostatus and the results remained very similar (Supplementary tables 14 and 15). Fourth, another important concern is the possibility of reverse causation, as the presence of GC at the time of diagnosis might have influenced participants’ exposure to some risk factors. Fifth, hospital-based controls may modify their diets due to underlying conditions, which could have artificially inflated the associations observed. Nevertheless, we performed sensitivity analyses and associations remained consistent independently of using hospital-based or population-based controls (Supplementary Table 13). Finally, the case–control design of the included studies is inherently more susceptible to bias than other prospective study designs, so our findings should be interpreted with caution.

This work however also has strengths. We collected dietary data from different countries, across various timeframes and with diverse study protocols, but they were harmonized by using a pre-specified format. This data pooling and harmonization provided us with a large and diverse sample for the main analysis, enough to observe significant associations with dose–response after adjusting for energy intake and major risk factors such as tobacco use, alcohol consumption and socioeconomic status. We also performed sensitivity analyses (Supplementary Table 12), in which we excluded each study one by one and found no changes in the associations with GC, which supports the robustness of our results. In addition, the large study sample allowed us to conduct adequately powered analyses by GC histological subtype, anatomical location, and sex; the sex-stratified analyses are particularly relevant given the well-known men predominance of GC and the limited evidence on whether dietary associations differ by sex. Finally, some associations showed a dose–response relationship providing further support and robustness for our key findings.

## Conclusion

Our study supports the hypotheses that adherence to gPVG and hPVG dietary patterns is inversely associated with the risk of GC, while adherence to the uPVG dietary pattern is associated with an increased GC risk. Sex-stratified analyses further revealed that the protective effect of hPVG was more pronounced in women, while the positive association of uPVG with GC risk was driven predominantly by women, suggesting that dietary patterns may play a particularly relevant role in GC prevention in this group. These results may help to design sex-sensitive preventive strategies to reduce the risk of GC.

## Supplementary Information

Below is the link to the electronic supplementary material.

Supplementary Material 1

## Abbreviations

GC: Gastric cancer
PVG: Pro-vegetarian
gPVG: General pro-vegetarian
hPVG: Healthful pro-vegetarian
uPVG: Unhealthful pro-vegetarian
StoP: Stomach cancer pooling
OR: Odds ratio
CI: Confidence interval
FFQ: Food frequency questionnaire
Q: Quintile
PDI: Plant-based dietary index
hPDI: Healthful plant-based dietary index
uPDI: Unhealthful plant-based dietary index
MD: Mediterranean diet
UPF: Ultra-processed food
