# Supplementary material for: Adherence to pro-vegetarian dietary patterns and gastric cancer risk: a pooled analysis of the StoP Consortium
Source: Gastric Cancer. 2026 May 14;29(4):715–24. doi: 10.1007/s10120-026-01757-4 (PMC13314681; doi:10.1007/s10120-026-01757-4)
Supplement: Supplementary file 1 — Supplementary Material 1 [file 10120_2026_1757_MOESM1_ESM.pdf]

Supplementary Material of this manuscript includes:

Tables:

Supplementary Table 1. Food items and scoring criteria for each pro-vegetarian dietary pattern in study 1 (Italy).

Supplementary Table 2. Food items and scoring criteria for each pro-vegetarian dietary pattern in study 2 (Greece).

Supplementary Table 3. Food items and scoring criteria for each pro-vegetarian dietary pattern in study 3 (Portugal).

Supplementary Table 4. Food items and scoring criteria for each pro-vegetarian dietary pattern in study 4 (Spain_1).

Supplementary Table 5. Food items and scoring criteria for each pro-vegetarian dietary pattern in study 5 (Spain_2).

Supplementary Table 6. Food items and scoring criteria for each pro-vegetarian dietary pattern in study 6 (México).

Supplementary Table 7. Quintile ranges for the three pro-vegetarian patterns in each study.

Supplementary Table 8. Odds ratios and 95% confidence intervals of gastric cancer according to the intake of main food groups considered in PVG dietary patterns in the StoP Consortium.

Supplementary table 9. Odds ratios and 95% confidence intervals of gastric cancer by histological subtype, according to adherence to pro-vegetarian dietary patterns (in quintiles, Q) in the StoP Consortium.

Supplementary Table 10. Odds ratios and 95% confidence intervals of adherence to pro-vegetarian dietary patterns (in quintiles, Q) and gastric cancer by anatomical subsites in the StoP Consortium.

Supplementary Table 11. Odds ratios and 95% confidence intervals of adherence to pro-vegetarian dietary patterns (in quintiles, Q) and gastric cancer by histological type and anatomical subsites in the StoP Consortium.

Supplementary Table 12. Sensitivity analysis: odds ratios and 95% confidence intervals for adherence to pro-vegetarian dietary patterns (in quintiles, Q) and gastric cancer risk after sequential exclusion of each study in the StoP Consortium.

Supplementary Table 13. Sensitivity analysis: odds ratios and 95% confidence intervals for adherence to pro-vegetarian dietary patterns (in quintiles, Q) and gastric cancer risk by type of control (hospital-based or population-based).

Supplementary table 14. Sensitivity analysis without missing data for Helicobacter pilory infection status: Odds ratios and 95% confidence intervals of gastric cancer according to the adherence to pro-vegetarian dietary patterns (in quintiles, Q) in the StoP Consortium.

Supplementary table 15. Stratified analysis for Helicobacter pilory infection status: Odds ratios and 95% confidence intervals of gastric cancer according to the adherence to pro-vegetarian dietary patterns (in quintiles, Q) in the StoP Consortium.

Figures:

Supplementary Figure 1. Association between selected food groups and gastric cancer risk in the StoP Consortium.

Supplementary Table 1. Food items and scoring criteria for each pro-vegetarian dietary pattern in study 1 (Italy, which FFQ comprised 78 items)[a].

**Food groups**	**Included food items**	**gPVG**[c]	**hPVG**	**uPVG**
Plant food groups[b]				
1. Vegetables	Corn, salad vegetables, raw carrots, cooked carrots, onions, artichokes, cruciferous vegetables (broccoli, cabbage, cauliflower), spinach and other leafy greens, tomatoes, salad with carrots, cucumbers, and peppers, cooked courgette/aubergines/peppers	Positive	Positive	Reverse
2. Fruits	Apples and pears, bananas, kiwi, cooked fruits, citrus fruits, peaches, apricots, plums, melon, grapes, strawberries and cherries	Positive	Positive	Reverse
3. Legumes	Peas/beans/chickpeas/lentils	Positive	Positive	Reverse
4. Whole grains	Whole-grain bread	Positive	Positive	Reverse
5. Refined grains	White bread, crackers, flatbreads, small rolls, pasta/rice with butter or oil, broth with noodles	Positive	Reverse	Positive
6. Potatoes	Boiled potatoes	Positive	Positive	Reverse
7. Fries and chips	Fried or roasted potatoes	Positive	Reverse	Positive
8. Nuts	-	Positive	Positive	Reverse
9. Olive oil	-	Positive	Positive	Reverse
10. Tea and coffee	Coffee, decaffeinated coffee, tea	Not scored	Positive	Reverse
11. Fruit juices	Natural fruit juice, packaged and sweetened fruit juice	Not scored	Reverse	Positive
12. Sugar-sweetened beverages	Carbonated soft drinks	Not scored	Reverse	Positive
13. Sweets and desserts	Sugar, honey and jams, biscuits, croissants and doughnuts, pastries, croissants with cream, cakes, fruit pies, chocolates, sweets	Not scored	Reverse	Positive
Animal food groups				
14. Meat/meat products	Boiled chicken/turkey, roast, fried or steamed chicken/turkey or rabbit, beef or horse steak, boiled beef, beef or veal meatballs, schnitzel steak, pork, liver, italian ham, ham, salami/mortadella/sausages/bacon/hot dog	Reverse	Reverse	Reverse
15. Animal fats for cooking or as a spread	-	Reverse	Reverse	Reverse
16. Eggs	Raw, poached or boiled eggs, fried eggs or omelette	Reverse	Reverse	Reverse
17. Fish and other seafood	Boiled fish and seafood, fried fish and seafood, tin of tuna/sardines in oil	Reverse	Reverse	Reverse
18. Dairy products	Whole milk, semi-skimmed milk, skimmed milk, yoghurt, grated cheese (parmesan), ricotta/mozzarella cheese, other cheeses, any other added cheese, ice cream	Reverse	Reverse	Reverse

Abbreviations: gPVG, general pro-vegetarian dietary pattern; hPVG, healthful pro-vegetarian dietary pattern; uPVG, unhealthful pro-vegetarian dietary pattern.
[a.] Positive indicates that higher consumption of this food group received higher scores. Reverse indicates that higher consumption of this food group received lower scores.
[b.] In the hPVG dietary pattern, whole grains, fruits, vegetables, nuts, legumes, potatoes (boiled), tea, and coffee were considered "healthy plant foods." Refined grains, fries or chips, fruit juices, sugar-sweetened beverages, and sweets and desserts were considered "unhealthy plant foods." The gPVG dietary pattern did not differentiate plant foods as healthy or unhealthy.
[c.] In the gPVG dietary pattern, consumption of whole grains and refined grains were considered as the "grains" group and boiled potatoes and fries or chips were considered as the "potatoes" group.

Supplementary Table 2. Food ítems and **s**coring criteria for each pro-vegetarian dietary pattern in study 2 (Greece, which FFQ comprised 80 items)[a].

**Food groups**	**Included food items**	**gPVG**[c]	**hPVG**	**uPVG**
Plant food groups[b]				
1. Vegetables	Tomatoes, cucumber, zucchini, onions, fresh beans, eggplants, cabbages, lettuce, spinach, leeks, chicory and endives, artichokes, beets, carrots, broccoli, cauliflower	Positive	Positive	Reverse
2. Fruits	Watermelons, melons, tangerines, lemons, oranges, apples, peaches, pears, grapes, apricots, cherries, bananas, figs, stewed fruit	Positive	Positive	Reverse
3. Legumes	Fresh broad beans, dried peas (kind of peas), peas, dry beans, chickpeas, lentils, dried broad beans, split peas	Positive	Positive	Reverse
4. Whole grains	-	Positive	Positive	Reverse
5. Refined grains	White bread, rye bread, pasta, rice	Positive	Reverse	Positive
6. Potatoes	Potatoes	Positive	Positive	Reverse
7. Fries and chips	-	Positive	Reverse	Positive
8. Nuts	Nuts (generally)	Positive	Positive	Reverse
9. Olive oil	Olive oil	Positive	Positive	Reverse
10. Tea and coffee	Coffee, tea	Not scored	Positive	Reverse
11. Fruit juices	-	Not scored	Reverse	Positive
12. Sugar-sweetened beverages	Cola	Not scored	Reverse	Positive
13. Sweets and desserts	Sugar, biscuits, chocolate, baked sweets with syrup, jelly, fruit glace, preserves	Not scored	Reverse	Positive
Animal food groups				
14. Meat/meat products	Pork, beef, lamp, goat, rabbit, chicken, processed meat, offal	Reverse	Reverse	Reverse
15. Animal fats for cooking or as a spread	Butter	Reverse	Reverse	Reverse
16. Eggs	Eggs	Reverse	Reverse	Reverse
17. Fish and other seafood	Fish	Reverse	Reverse	Reverse
18. Dairy products	Cheese, milk, yogurt, ice cream	Reverse	Reverse	Reverse

Abbreviations: gPVG, general pro-vegetarian dietary pattern; hPVG, healthful pro-vegetarian dietary pattern; uPVG, unhealthful pro-vegetarian dietary pattern.

[a.] Positive indicates that higher consumption of this food group received higher scores. Reverse indicates that higher consumption of this food group received lower scores.

[b.] In the hPVG dietary pattern, whole grains, fruits, vegetables, nuts, legumes, potatoes (boiled), tea, and coffee were considered “healthy plant foods.” Refined grains, fries or chips, fruit juices, sugar-sweetened beverages, and sweets and desserts were considered “unhealthy plant foods.” The gPVG dietary pattern did not differentiate plant foods as healthy or unhealthy.

[c.] In the gPVG dietary pattern, consumption of whole grains and refined grains were considered as the “grains” group and boiled potatoes and fries or chips were considered as the “potatoes” group.

Supplementary Table 3. Food ítems and scoring criteria for each pro-vegetarian dietary pattern in study 3 (Portugal, which FFQ comprised 82 items)[a].

Food groups	Included food items	gPVG[c]	hPVG	uPVG
Plant food groups[b]				
1. Vegetables	Cabbage, spring greens, kale, broccoli, cauliflower, brussels sprouts, turnip greens, spinach, green beans, lettuce, watercress, onion, carrot, turnip, tomato, sweet pepper, cucumber	Positive	Positive	Reverse
2. Fruits	Apple, pear, orange, tangerine, banana, kiwi, strawberries, cherries, peach, plum, melon, watermelon, persimmon, fig, medlar, apricot, grapes, canned fruits: peach, pineapple	Positive	Positive	Reverse
3. Legumes	Beans, chickpea, peas, broad beans	Positive	Positive	Reverse
4. Whole grains	Brown or whole wheat bread or toasts	Positive	Positive	Reverse
5. Refined grains	White bread or toasts, corn bread ‘broa’, rice, pasta	Positive	Reverse	Positive
6. Potatoes	Boiled, baked or stewed potatoes	Positive	Positive	Reverse
7. Fries and chips	Potato chips	Positive	Reverse	Positive
8. Nuts	Dried fruits: almonds, hazelnuts, peanuts	Positive	Positive	Reverse
9. Olive oil	Olive oil	Positive	Positive	Reverse
10. Tea and coffee	Coffee, black tea	Not scored	Positive	Reverse
11. Fruit juices	-	Not scored	Reverse	Positive
12. Sugar-sweetened beverages	Soft drinks, Coca-cola	Not scored	Reverse	Positive
13. Sweets and desserts	Other cookies/biscuits, croissant, pastry or cake, chocolate bar or powder, marmelade, jam, honey, sugar	Not scored	Reverse	Positive
Animal food groups				
14. Meat/meat products	Chicken, turkey, rabbit, beef, pork, lamb, beef, pork or chicken liver, offals, ham, pudding, sausages, bacon	Reverse	Reverse	Reverse
15. Animal fats for cooking or as a spread	Butter	Reverse	Reverse	Reverse
16. Eggs	Eggs	Reverse	Reverse	Reverse
17. Fish and other seafood	Oily fish, white fish, codfish, canned fish (tuna, sardines), squid, octopus, shell fish	Reverse	Reverse	Reverse
18. Dairy products	Whole milk, semi-skimed milk, skim milk, yogurt, cheese, dairy deserts, ice cream	Reverse	Reverse	Reverse

Abbreviations: gPVG, general pro-vegetarian dietary pattern; hPVG, healthful pro-vegetarian dietary pattern; uPVG, unhealthful pro-vegetarian dietary pattern.

[a.] Positive indicates that higher consumption of this food group received higher scores. Reverse indicates that higher consumption of this food group received lower scores.

[b.] In the hPVG dietary pattern, whole grains, fruits, vegetables, nuts, legumes, potatoes (boiled), tea, and coffee were considered “healthy plant foods.” Refined grains, fries or chips, fruit juices, sugar-sweetened beverages, and sweets and desserts were considered “unhealthy plant foods.” The gPVG dietary pattern did not differentiate plant foods as healthy or unhealthy.

[c.] In the gPVG dietary pattern, consumption of whole grains and refined grains were considered as the “grains” group and boiled potatoes and fries or chips were considered as the “potatoes” group.

Supplementary Table 4. Food ítems and **s**coring criteria for each pro-vegetarian dietary pattern in study 4 (Spain_1, which FFQ comprised 140 items)[a].

**Food groups**	**Included food items**	**gPVG**[c]	**hPVG**	**uPVG**
Plant food groups[b]				
1. Vegetables	Lettuce, leafy greens, tomato, cucumber, onion, radish, beetroot, asparagus, carrots, sweetcorn, artichokes, pumpkin, gazpacho, sweet potato, leeks, other vegetables, vegetable purée, green beans, spinach, chard, cabbage, broccoli, aubergine, red peppers, green peppers	Positive	Positive	Reverse
2. Fruits	Oranges, tangerines, banana, apple, pear, grapes, kiwi, strawberries, cherries, peach, apricot, peach, pear in syrup, dried fruits, other fresh fruit, figs, watermelon, melon, plum, mango, papaya	Positive	Positive	Reverse
3. Legumes	Peas, lentils, chickpeas and beans, white beans, broad beans	Positive	Positive	Reverse
4. Whole grains	Wholegrain bread, wholegrain cereals, wholegrain breadsticks, rolls	Positive	Positive	Reverse
5. Refined grains	White bread, regular cereals, breadsticks, rolls, rice, pasta	Positive	Reverse	Positive
6. Potatoes	Boiled potatoes, pureé	Positive	Positive	Reverse
7. Fries and chips	Fried potatoes (no chips)	Positive	Reverse	Positive
8. Nuts	Nuts	Positive	Positive	Reverse
9. Olive oil	Olive oil	Positive	Positive	Reverse
10. Tea and coffee	Caffeinated coffee, decaffeinated coffee	Not scored	Positive	Reverse
11. Fruit juices	Orange juice, fresh juice, packaged juice	Not scored	Reverse	Positive
12. Sugar-sweetened beverages	Regular soft drinks, diet soft drinks	Not scored	Reverse	Positive
13. Sweets and desserts	Maria biscuit, wholegrain Maria biscuit, chocolate biscuits, pastries: croissant, donut, cupcake, sponge cake, cake, tart, churro, fritter, chocolate, bonbon, cocoa powder, nougat, shortbread, christmas sweets, jam, preserves, sugar	Not scored	Reverse	Positive
Animal food groups				
14. Meat/meat products	Duck fat, pork meatballs, beef meatballs, rabbit, hare, beef or chicken liver, offal, bacon, lard, cooked ham, cured ham, fuet, salchichón, other cold cuts, frankfurter, similar, pâté, cured beef, botillo, other smoked cold meats	Reverse	Reverse	Reverse
15. Animal fats for cooking or as a spread	Butter, lard	Reverse	Reverse	Reverse
16. Eggs	Eggs	Reverse	Reverse	Reverse
17. Fish and other seafood	Mussels, clams, octopus, squid, prawns, king prawns, salted fish, canned fish, canned seafood, smoked fish	Reverse	Reverse	Reverse
18. Dairy products	Skimmed/semi-skimmed milk, whole milk, condensed milk, low-fat yoghurt, whole yoghurt, cottage cheese, mató, fresh white cheese, portion cream cheese, cured/semi-cured cheese, blue cheese, roquefort, custards, flan, cream, milkshake, creamy ice cream, light creamy ice cream	Reverse	Reverse	Reverse

Abbreviations: gPVG, general pro-vegetarian dietary pattern; hPVG, healthful pro-vegetarian dietary pattern; uPVG, unhealthful pro-vegetarian dietary pattern.

[a.] Positive indicates that higher consumption of this food group received higher scores. Reverse indicates that higher consumption of this food group received lower scores.

[b.] In the hPVG dietary pattern, whole grains, fruits, vegetables, nuts, legumes, potatoes (boiled), tea, and coffee were considered "healthy plant foods." Refined grains, fries or chips, fruit juices, sugar-sweetened beverages, and sweets and desserts were considered "unhealthy plant foods." The gPVG dietary pattern did not differentiate plant foods as healthy or unhealthy.

[c.] In the gPVG dietary pattern, consumption of whole grains and refined grains were considered as the "grains" group and boiled potatoes and fries or chips were considered as the "potatoes" group.

Supplementary Table 5. Food ítems and **s**coring criteria for each pro-vegetarian dietary pattern in study 5 (Spain_2, which FFQ comprised 93 items)[a].

**Food groups**	**Included food items**	**gPVG**[c]	**hPVG**	**uPVG**
Plant food groups[b]				
1. Vegetables	Spinach, cabbage, cauliflower, broccoli, lettuce, endive, tomatoes, onion, carrot, pumpkin, green beans, eggplant, zucchini, cucumber, peppers, asparagus	Positive	Positive	Reverse
2. Fruits	Oranges, grapefruit, mandarin, banana, apple, pear, strawberries, cherries, peaches, apricots, fresh figs, watermelon, melon, grapes, canned fruit (peach, pear, pineapple)	Positive	Positive	Reverse
3. Legumes	Lentils, chickpeas, beans, peas	Positive	Positive	Reverse
4. Whole grains	Whole-grain bread	Positive	Positive	Reverse
5. Refined grains	White bread, rolls, white rice, white pasta	Positive	Reverse	Positive
6. Potatoes	Boiled and roasted potatoes	Positive	Positive	Reverse
7. Fries and chips	French fries, potato chips	Positive	Reverse	Positive
8. Nuts	Pine nuts, almonds, peanuts, hazelnuts, and other nuts	Positive	Positive	Reverse
9. Olive oil	Olive oil	Positive	Positive	Reverse
10. Tea and coffee	Caffeinated coffee, decaffeinated coffee, tea	Not scored	Positive	Reverse
11. Fruit juices	Orange juice, other package fruit juices	Not scored	Reverse	Positive
12. Sugar-sweetened beverages	Carbonated soft drinks: cola, orange, lemon	Not scored	Reverse	Positive
13. Sweets and desserts	Maria cookies, chocolate cookies, croissants, donuts, muffins, cakes, pies, churros (fried dough), chocolate, bonbons, cocoa powder, sugar	Not scored	Reverse	Positive
Animal food groups				
14. Meat/meat products	Chicken with or without skin, beef, pork, lamb, game meat (rabbit, quail, duck), liver of beef, pork or chicken, viscera, cold cuts (ham, salami, mortadella) sausages and similar, foie gras, hamburger, bacon	Reverse	Reverse	Reverse
15. Animal fats for cooking or as a spread	Butter, lard	Reverse	Reverse	Reverse
16. Eggs	Eggs	Reverse	Reverse	Reverse
17. Fish and other seafood	Fried fish, boiled or grilled fish (hake, sole, sardines, tuna), salted fish (cod, anchovies), canned fish (tuna, sardines, herring), clams, mussels, oysters, squid, octopus, shellfish (prawns, lobster and similar)	Reverse	Reverse	Reverse
18. Dairy products	Whole milk, skim or low-fat milk, condensed milk, yoghurt, cottage cheese, curd, white or fresh cheese, creamy cheese or cheese in portions, cured or semi-cured cheese (Manchego), custard, flan, pudding, ice cream	Reverse	Reverse	Reverse

Abbreviations: gPVG, general pro-vegetarian dietary pattern; hPVG, healthful pro-vegetarian dietary pattern; uPVG, unhealthful pro-vegetarian dietary pattern.

[a.] Positive indicates that higher consumption of this food group received higher scores. Reverse indicates that higher consumption of this food group received lower scores.

[b.] In the hPVG dietary pattern, whole grains, fruits, vegetables, nuts, legumes, potatoes (boiled), tea, and coffee were considered “healthy plant foods.” Refined grains, fries or chips, fruit juices, sugar-sweetened beverages, and sweets and desserts were considered “unhealthy plant foods.” The gPVG dietary pattern did not differentiate plant foods as healthy or unhealthy.

[c.] In the gPVG dietary pattern, consumption of whole grains and refined grains were considered as the “grains” group and boiled potatoes and fries or chips were considered as the “potatoes” group.

Supplementary Table 6. Food ítems and scoring criteria for each pro-vegetarian dietary pattern in study 6 (México, which FFQ comprised 127 items)[a].

**Food groups**	**Included food items**	**gPVG**[c]	**hPVG**	**uPVG**
Plant food groups[b]				
1. Vegetables	Cauliflower, broccoli, purslane, corn, carrots, spinach, zucchini, squash, lettuce, cooked tomato, raw tomato, nopales, squash blossom, beet, onion, garlic	Positive	Positive	Reverse
2. Fruits	Banana, plums, peach, apple, orange, grapes, blackberries, strawberries, cantaloupe, watermelon, mango, tangerine, pear, mammy-apple, tuna, sapodilla, papaya, pineapple	Positive	Positive	Reverse
3. Legumes	Beans, peas, lentils, lima beans	Positive	Positive	Reverse
4. Whole grains	-	Positive	Positive	Reverse
5. Refined grains	Corn tortilla, wheat flour tortilla, bread, bolillo, sweet bread, rice, corn flakes	Positive	Reverse	Positive
6. Potatoes	Potatoe or yam	Positive	Positive	Reverse
7. Fries and chips	-	Positive	Reverse	Positive
8. Nuts	-	Positive	Positive	Reverse
9. Olive oil	Olive oil	Positive	Positive	Reverse
10. Tea and coffee	Brewed black coffee, instant coffee, black tea, herbal tea	Not scored	Positive	Reverse
11. Fruit juices	Orange juice	Not scored	Reverse	Positive
12. Sugar-sweetened beverages	Soda	Not scored	Reverse	Positive
13. Sweets and desserts	Cake, cracker	Not scored	Reverse	Positive
Animal food groups				
14. Meat/meat products	Chicken, bacon, sausage, ham, liver steak, chorizo, beef, pork meat, jerky beef, pork cracklings, barbacoa, carnitas	Reverse	Reverse	Reverse
15. Animal fats for cooking or as a spread	Butter, lard	Reverse	Reverse	Reverse
16. Eggs	Eggs	Reverse	Reverse	Reverse
17. Fish and other seafood	Tuna, sardine, fresh fish, seafood	Reverse	Reverse	Reverse
18. Dairy products	Whole milk, cream cheese, oaxaca cheese, manchego cheese, sour cream, ice cream, yoghurt, fresh cheese	Reverse	Reverse	Reverse

Abbreviations: gPVG, general pro-vegetarian dietary pattern; hPVG, healthful pro-vegetarian dietary pattern; uPVG, unhealthful pro-vegetarian dietary pattern.

[a.] Positive indicates that higher consumption of this food group received higher scores. Reverse indicates that higher consumption of this food group received lower scores.

[b.] In the hPVG dietary pattern, whole grains, fruits, vegetables, nuts, legumes, potatoes (boiled), tea, and coffee were considered "healthy plant foods." Refined grains, fries or chips, fruit juices, sugar-sweetened beverages, and sweets and desserts were considered "unhealthy plant foods." The gPVG dietary pattern did not differentiate plant foods as healthy or unhealthy.

[c.] In the gPVG dietary pattern, consumption of whole grains and refined grains were considered as the "grains" group and boiled potatoes and fries or chips were considered as the "potatoes" group.

Supplementary Table 7. Quintile ranges for the three pro-vegetarian patterns in each study.

Study country	gPVG quintiles rank				
	Q1	Q2	Q3	Q4	Q5
Study 1_Italy	<37	37-39	40-41	42-44	≥45
Study 2_Greece	<35	35-36	37-39	40-42	≥43
Study 3_Portugal	<39	39-41	42-43	44-46	≥47
Study 4_Spain_1	<38	38-41	42-43	44-47	≥48
Study 5_Spain_2	<38	38-40	41-43	44-47	≥48
Study 6_Mexico	<31	31-33	34-35	36-38	≥39
	**hPVG quintiles rank**				
	**Q1**	**Q2**	**Q3**	**Q4**	**Q5**
Study 1_Italy	<48	48-50	51-54	55-57	≥58
Study 2_Greece	<52	52-54	55-57	58-61	≥62
Study 3_Portugal	<51	51-54	55-58	59-62	≥63
Study 4_Spain_1	<49	49-52	53-56	57-60	≥61
Study 5_Spain_2	<49	49-52	53-56	57-60	≥61
Study 6_Mexico	<46	46-49	50-51	52-54	≥55
	**uPVG quintiles rank**				
	**Q1**	**Q2**	**Q3**	**Q4**	**Q5**
Study 1_Italy	<56	56-58	59-61	62-66	≥67
Study 2_Greece	<49	49-51	52-54	55-56	≥57
Study 3_Portugal	<47	47-50	51-54	55-58	≥59
Study 4_Spain_1	<49	49-52	53-56	57-60	≥61
Study 5_Spain_2	<50	50-53	54-56	57-59	≥60
Study 6_Mexico	<54	54-57	58-59	60-63	≥64

Abbreviations: gPVG, general pro-vegetarian food pattern; hPVG, healthful pro-vegetarian food pattern; uPVG, unhealthful pro-vegetarian food pattern.

Supplementary table 8. Odds ratios and 95% confidence intervals of gastric cancer according to the intake of main food groups considered in PVG dietary patterns in the StoP Consortium.

Food groups	Q1	Q2	Q3	Q4	Q5	*p*-trend
Vegetables	1 (Ref.)	0.79 (0.66-0.94)	0.77 (0.64-0.92)	0.65 (0.53-0.78)	0.58 (0.47-0.71)	<0.001
	1 (Ref.)	0.85 (0.72-1.01)	0.72 (0.60-0.86)	0.61 (0.51-0.74)	0.52 (0.42-0.63)	<0.001
Legumes	1 (Ref.)	0.93 (0.78-1.11)	1.08 (0.89-1.32)	1.14 (0.93-1.39)	1.08 (0.88-1.32)	0.009
	1 (Ref.)	0.95 (0.78-1.16)	1.06 (0.87-1.29)	1.12 (0.92-1.37)	1.06 (0.87-1.30)	0.281
Whole grains	1 (Ref.)	1.32 (1.07-1.63)	1.38 (1.15-1.66)	1.37 (1.13-1.66)	0.86 (0.70-1.06)	<0.001
Refined grains	1 (Ref.)	1.20 (0.97-1.48)	1.25 (1.02-1.54)	1.38 (1.12-1.70)	1.34 (1.09-1.65)	0.004
	1 (Ref.)	1.27 (1.04-1.53)	1.19 (0.97-1.46)	1.34 (1.08-1.66)	1.20 (0.96-1.50)	0.063
Boiled potatoes	1 (Ref.)	1.23 (1.02-1.49)	1.23 (1.01-1.50)	1.31 (1.06-1.61)	1.65 (1.32-2.07)	<0.001
Fried potatoes	1 (Ref.)	0.96 (0.77-1.21)	0.70 (0.57-0.87)	0.79 (0.64-0.98)	0.59 (0.47-0.73)	<0.001
	1 (Ref.)	1.31 (0.97-1.76)	1.32 (1.08-1.62)	0.83 (0.68-1.02)	0.85 (0.69-1.06)	0.052
Olive oil	1 (Ref.)	0.84 (0.59-1.17)	1.02 (0.72-1.44)	0.80 (0.56-1.14)	0.66 (0.46-0.95)	0.003
Tea and coffee	1 (Ref.)	0.77 (0.64-0.92)	0.76 (0.63-0.92)	0.73 (0.61-0.88)	0.68 (0.57-0.83)	0.003
Fruit juices	1 (Ref.)	—	0.88 (0.70-1.11)	1.04 (0.85-1.28)	1.15 (0.93-1.42)	0.082
sweetened beverages	1 (Ref.)	1.20 (0.99-1.45)	1.06 (0.87-1.29)	1.02 (0.84-1.23)	1.26 (1.04-1.52)	0.107
Sweets and desserts	1 (Ref.)	1.10 (0.90-1.35)	1.13 (0.92-1.39)	1.27 (1.03-1.57)	1.21 (0.98-1.50)	0.009
Meat/meat products	1 (Ref.)	1.48 (1.17-1.88)	1.65 (1.27-2.15)	1.82 (1.37-2.43)	1.79 (1.34-2.40)	0.035
Animal fats	1 (Ref.)	1.00 (0.79-1.26)	1.20 (0.97-1.49)	1.13 (0.90-1.41)	0.98 (0.80-1.19)	0.001
	1 (Ref.)	1.12 (0.93-1.34)	1.10 (0.90-1.36)	0.91 (0.76-1.10)	1.21 (1.01-1.46)	0.025
Fish and other seafood	1 (Ref.)	0.91 (0.75-1.10)	0.79 (0.64-0.97)	0.87 (0.70-1.08)	0.91 (0.72-1.14)	0.712
Dairy products	1 (Ref.)	0.94 (0.79-1.12)	1.15 (0.96-1.37)	1.18 (0.98-1.42)	1.06 (0.88-1.27)	0.077

: Q2 was merged into Q1 owing to an excess of zero-consumption values, making it impossible to define five independent quintiles.

adjusted for sex (men; women), age (<49; 50-59; 60-69; ≥70 years), social class (low; intermediate; high), tobacco smoking (never; former; current), alcohol consumption (0; 1-2, ≥3 drinks/day), energy intake (kcal/day) and *Helicobacter pylori* infection (seronegative, seropositive, missing).

Supplementary table 9. Odds ratios and 95% confidence intervals of gastric cancer by histological subtype, according to adherence to pro-vegetarian dietary patterns (in quintiles, Q) in the StoP Consortium.

	**gPVG dietary pattern**						
	**Q1** (<37)[a]	**Q2** (37-40)[a]	**Q3** (41-43)[a]	**Q4** (44-46)[a]	**Q5** (≥47)[a]	**Per 1 quintile increment**	**Per 5 points increment in adherence**
Intestinal (*n*=730)	1 (Ref.)	0.82 (0.64-1.06)	0.75 (0.56-1.01)	0.69 (0.52-0.90)	0.65 (0.49-0.86)	0.90 (0.84-0.96)	0.87 (0.80-0.94)
Diffuse (*n*=507)	1 (Ref.)	0.82 (0.63-1.08)	0.82 (0.59-1.14)	0.66 (0.48-0.90)	0.46 (0.31-0.66)	0.85 (0.78-0.91)	0.80 (0.73-0.88)
Other type (*n*=206)	1 (Ref.)	1.04 (0.66-1.63)	0.74 (0.44-1.27)	1.16 (0.74-1.82)	0.65 (0.38-1.11)	0.94 (0.85-1.05)	0.91 (0.79-1.05)
	**hPVG dietary pattern**						
	**Q1** (<49)[1]	**Q2** (49-52)[1]	**Q3** (53-56)[1]	**Q4** (57-60)[1]	**Q5** (≥61)[1]	**Per 1 quintile increment**	**Per 5 points increment in adherence**
Intestinal (*n*=730)	1 (Ref.)	0.86 (0.66-1.13)	0.97 (0.75-1.25)	0.98 (0.75-1.28)	0.73 (0.55-0.97)	0.95 (0.89-1.01)	0.94 (0.88-1.00)
Diffuse (*n*=507)	1 (Ref.)	0.89 (0.68-1.17)	0.73 (0.55-0.96)	0.69 (0.51-0.95)	0.46 (0.32-0.66)	0.84 (0.78-0.91)	0.82 (0.76-0.88)
Other type (*n*=206)	1 (Ref.)	1.21 (0.76-1.92)	1.04 (0.65-1.65)	0.97 (0.59-1.58)	1.00 (0.61-1.64)	0.97 (0.87-1.09)	0.97 (0.87-1.09)
	**uPVG dietary pattern**						
	**Q1** (<49)[1]	**Q2** (49-53)[1]	**Q3** (54-56)[1]	**Q4** (57-60)[1]	**Q5** (≥61)[1]	**Per 1 quintile increment**	**Per 5 points increment in adherence**
Intestinal (*n*=730)	1 (Ref.)	1.18 (0.91-1.53)	1.37 (1.04-1.80)	1.24 (0.95-1.64)	1.04 (0.78-1.40)	1.01 (0.95-1.08)	1.00 (0.94-1.07)
Diffuse (*n*=507)	1 (Ref.)	1.10 (0.79-1.53)	1.12 (0.79-1.61)	1.45 (1.04-2.01)	1.44 (1.03-2.00)	1.10 (1.03-1.19)	1.11 (1.03-1.19)
Other type (*n*=206)	1 (Ref.)	1.36 (0.86-2.15)	1.02 (0.61-1.72)	1.00 (0.60-1.68)	1.29 (0.78-2.11)	1.02 (0.91-1.14)	1.03 (0.92-1.15)

Abbreviations: gPVG, general pro–vegetarian dietary pattern; hPVG, healthful pro–vegetarian dietary pattern; uPVG, unhealthful pro–vegetarian dietary pattern.

[a] Adherence range points.

Model adjusted for sex (men; women), age (<49; 50-59; 60-69; ≥70 years), social class (low; intermediate; high), tobacco smoking (never; former; current), alcohol consumption (0; 1-2, ≥3 drinks/day), energy intake (kcal/day) and *Helicobacter pylori* infection (seronegative, seropositive, missing).

Supplementary Table 10. Odds ratios and 95% confidence intervals of adherence to pro-vegetarian dietary patterns (in quintiles, Q) and gastric cancer by anatomical subsites in the StoP Consortium.

	**gPVG dietary pattern**						
	**Q1** (<37)[a]	**Q2** (37-40)	**Q3** (41-43)	**Q4** (44-46)	**Q5** (≥47)	**Per 1 quintile increment**	**Per 5 points increment in adherence**
Cardia (*n*=170)	1 (Ref.)	0.85 (0.53-1.36)	0.73 (0.45-1.19)	0.71 (0.42-1.19)	0.54 (0.31-0.92)	0.87 (0.77-0.98)	0.87 (0.75-1.01)
Non-cardia (*n*=1,313)	1 (Ref.)	0.78 (0.63-0.93)	0.75 (0.60-0.94)	0.63 (0.51-0.77)	0.57 (0.45-0.71)	0.87 (0.83-0.92)	0.84 (0.79-0.89)
	**hPVG dietary pattern**						
	**Q1** (<49)[a]	**Q2** (49-52)	**Q3** (53-56)	**Q4** (57-60)	**Q5** (≥61)	**Per 1 quintile increment**	**Per 5 points increment in adherence**
Cardia (*n*=170)	1 (Ref.)	0.69 (0.43-1.13)	0.76 (0.49-1.19)	0.50 (0.30-0.85)	0.62 (0.39-1.01)	0.88 (0.78-0.99)	0.86 (0.76-0.96)
Non-cardia (*n*=1,313)	1 (Ref.)	0.89 (0.73-1.08)	0.81 (0.66-0.98)	0.81 (0.66-1.00)	0.62 (0.50-0.78)	0.90 (0.86-0.95)	0.89 (0.84-0.93)
	**uPVG dietary pattern**						
	**Q1** (<49)[a]	**Q2** (49-53)	**Q3** (54-56)	**Q4** (57-60)	**Q5** (≥61)	**Per 1 quintile increment**	**Per 5 points increment in adherence**
Cardia (*n*=170)	1 (Ref.)	1.36 (0.81-2.28)	1.56 (0.90-2.70)	1.49 (0.87-2.57)	1.86 (1.09-3.18)	1.14 (1.01-1.27)	1.13 (1.01-1.27)
Non-cardia (*n*=1,313)	1 (Ref.)	1.09 (0.90-1.33)	1.17 (0.94-1.45)	1.21 (0.98-1.50)	1.17 (0.94-1.46)	1.04 (0.99-1.09)	1.04 (0.99-1.09)

Abbreviations: gPVG, general pro-vegetarian dietary pattern; hPVG, healthful pro-vegetarian dietary pattern; uPVG, unhealthful pro-vegetarian dietary pattern.
[a]Adherence range points.
Model adjusted for sex (men; women), age (<49; 50-59; 60-69; ≥70 years), social class (low; intermediate; high), tobacco smoking (never; former; current), alcohol consumption (0; 1-2, ≥3 drinks/day), energy intake (kcal/day) and *Helicobacter pylori* infection (seronegative, seropositive, missing).

Supplementary Table 11. Odds ratios and 95% confidence intervals of adherence to pro-vegetarian dietary patterns (in quintiles, Q) and gastric cancer by histological type and anatomical subsites in the StoP Consortium.

	**gPVG dietary pattern**						
	**Q1** (<37)[a]	**Q2** (37-40)	**Q3** (41-43)	**Q4** (44-46)	**Q5** (≥47)	**Per 1 quintile increment**	**Per 5 points increment in adherence**
Intestinal, non-cardia (*n*=546)	1 (Ref.)	0.78 (0.59-1.05)	0.75 (0.54-1.04)	0.63 (0.47-0.86)	0.64 (0.46-0.88)	0.89 (0.83-0.96)	0.87 (0.80-0.96)
Diffuse, non-cardia (*n*=373)	1 (Ref.)	0.74 (0.54-1.00)	0.74 (0.51-1.07)	0.56 (0.39-0.80)	0.40 (0.26-0.62)	0.82 (0.75-0.89)	0.76 (0.68-0.85)
	**hPVG dietary pattern**						
	**Q1** (<49)[a]	**Q2** (49-52)	**Q3** (53-56)	**Q4** (57-60)	**Q5** (≥61)	**Per 1 quintile increment**	**Per 5 points increment in adherence**
Intestinal, non-cardia (*n*=546)	1 (Ref.)	0.90 (0.67-1.23)	1.02 (0.76-1.36)	1.10 (0.81-1.50)	0.81 (0.58-1.11)	0.98 (0.91-1.05)	0.97 (0.90-1.04)
Diffuse, non-cardia (*n*=373)	1 (Ref.)	0.86 (0.64-1.16)	0.64 (0.47-0.88)	0.66 (0.46-0.94)	0.42 (0.28-0.63)	0.82 (0.75-0.89)	0.80 (0.73-0.87)
	**uPVG dietary pattern**						
	**Q1** (<49)[a]	**Q2** (49-53)	**Q3** (54-56)	**Q4** (57-60)	**Q5** (≥61)	**Per 1 quintile increment**	**Per 5 points increment in adherence**
Intestinal, non-cardia (*n*=546)	1 (Ref.)	1.10 (0.83-1.47)	1.24 (0.92-1.67)	1.06 (0.78-1.44)	0.86 (0.62-1.21)	0.97 (0.90-1.04)	0.97 (0.90-1.04)
Diffuse, non-cardia (*n*=373)	1 (Ref.)	1.02 (0.70-1.47)	1.06 (0.71-1.58)	1.44 (1.00-2.08)	1.41 (0.97-2.04)	1.11 (1.02-1.21)	1.10 (1.01-1.20)

Abbreviations: gPVG, general pro-vegetarian dietary pattern; hPVG, healthful pro-vegetarian dietary pattern; uPVG, unhealthful pro-vegetarian dietary pattern.
[a]Adherence range points.
Model adjusted for sex (men; women), age (<49; 50-59; 60-69; ≥70 years), social class (low; intermediate; high), tobacco smoking (never; former; current), alcohol consumption (0; 1-2, ≥3 drinks/day), energy intake (kcal/day) and *Helicobacter pylori* infection (seronegative, seropositive, missing).

Supplementary Table 12. Sensitivity analysis: odds ratios and 95% confidence intervals for adherence to pro-vegetarian dietary patterns (in quintiles, Q) and gastric cancer risk after sequential exclusion of each study in the StoP Consortium.

	**gPVG dietary pattern**				
**Excluded studies**	**Q1**	**Q2**	**Q3**	**Q4**	**Q5**
Study 1_Italy (*n*=6759)	1 (Ref.)	0.77 (0.64-0.93)	0.76 (0.61-0.94)	0.64 (0.53-0.78)	0.56 (0.45-0.69)
Study 2_Greece (*n*=7298)	1 (Ref.)	0.81 (0.68-0.97)	0.80 (0.65-0.98)	0.70 (0.58-0.85)	0.62 (0.51-0.76)
Study 3_Portugal (*n*=5476)	1 (Ref.)	0.76 (0.62-0.92)	0.85 (0.67-1.08)	0.67 (0.54-0.84)	0.50 (0.39-0.64)
Study 4_Spain_1 (*n*=4512)	1 (Ref.)	0.81 (0.67-0.98)	0.76 (0.60-0.95)	0.73 (0.59-0.90)	0.60 (0.48-0.76)
Study 5_Spain_2 (*n*=6655)	1 (Ref.)	0.79 (0.66-0.95)	0.80 (0.65-1.00)	0.69 (0.57-0.84)	0.69 (0.55-0.86)
Study 6_México (*n*=6815)	1 (Ref.)	0.89 (0.74-1.07)	0.84 (0.68-1.03)	0.75 (0.62-0.91)	0.66 (0.53-0.81)
	**hPVG dietary pattern**				
	**Q1**	**Q2**	**Q3**	**Q4**	**Q5**
Study 1_Italy (*n*=6759)	1 (Ref.)	0.86 (0.71-1.04)	0.84 (0.70-1.01)	0.81 (0.66-0.99)	0.65 (0.53-0.80)
Study 2_Greece (*n*=7298)	1 (Ref.)	0.91 (0.76-1.08)	0.88 (0.74-1.04)	0.83 (0.69-1.00)	0.69 (0.56-0.84)
Study 3_Portugal (*n*=5476)	1 (Ref.)	0.85 (0.70-1.04)	0.78 (0.64-0.94)	0.59 (0.47-0.74)	0.50 (0.39-0.64)
Study 4_Spain_1 (*n*=4512)	1 (Ref.)	0.97 (0.79-1.18)	0.88 (0.72-1.07)	0.88 (0.71-1.09)	0.66 (0.52-0.82)
Study 5_Spain_2 (*n*=6655)	1 (Ref.)	0.87 (0.72-1.05)	0.80 (0.66-0.96)	0.80 (0.65-0.98)	0.69 (0.56-0.85)
Study 6_México (*n*=6815)	1 (Ref.)	1.02 (0.84-1.23)	0.93 (0.77-1.12)	0.84 (0.69-1.02)	0.74 (0.60-0.91)
	**uPVG dietary pattern**				
	**Q1**	**Q2**	**Q3**	**Q4**	**Q5**
Study 1_Italy (*n*=6759)	1 (Ref.)	1.14 (0.95-1.37)	1.22 (1.00-1.49)	1.22 (1.00-1.49)	1.19 (0.97-1.47)
Study 2_Greece (*n*=7298)	1 (Ref.)	1.16 (0.97-1.40)	1.24 (1.01-1.51)	1.21 (1.00-1.48)	1.29 (1.05-1.57)
Study 3_Portugal (*n*=5476)	1 (Ref.)	1.06 (0.83-1.35)	1.19 (0.93-1.54)	1.31 (1.03-1.68)	1.49 (1.16-1.91)
Study 4_Spain_1 (*n*=4512)	1 (Ref.)	1.12 (0.91-1.37)	1.26 (1.01-1.57)	1.28 (1.03-1.59)	1.26 (1.01-1.57)
Study 5_Spain_2 (*n*=6655)	1 (Ref.)	1.19 (0.98-1.46)	1.24 (1.00-1.54)	1.22 (0.99-1.51)	1.25 (1.01-1.54)
Study 6_México (*n*=6815)	1 (Ref.)	1.08 (0.89-1.30)	1.17 (0.95-1.43)	1.11 (0.90-1.35)	1.23 (1.00-1.52)

Abbreviations: gPVG, general pro-vegetarian dietary pattern; hPVG, healthful pro-vegetarian dietary pattern; uPVG, unhealthful pro-vegetarian dietary pattern.

Model adjusted for sex (men; women), age (<49; 50-59; 60-69; ≥70 years), social class (low; intermediate; high), tobacco smoking (never; former; current), alcohol consumption (0; 1-2, ≥3 drinks/day), energy intake (kcal/day) and *Helicobacter pylori* infection (seronegative, seropositive, missing).

Supplementary Table 13. Sensitivity analysis: odds ratios and 95% confidence intervals for adherence to pro-vegetarian dietary patterns (in quintiles, Q) and gastric cancer risk by type of control (hospital-based or population-based).

	**Hospital-based controls (*n*=1077)**				
	**Q1**	**Q2**	**Q3**	**Q4**	**Q5**
gPVG	1 (Ref.)	0.86 (0.65-1.14)	0.85 (0.63-1.15)	0.86 (0.61-1.21)	0.48 (0.34-0.69)
hPVG	1 (Ref.)	1.13 (0.84-1.52)	0.88 (0.65-1.18)	0.60 (0.43-0.84)	0.46 (0.32-0.66)
uPVG	1 (Ref.)	0.83 (0.58-1.18)	1.16 (0.80-1.68)	1.31 (0.91-1.88)	1.68 (1.14-2.47)
	**Population-based controls (*n*=4569)**				
	**Q1**	**Q2**	**Q3**	**Q4**	**Q5**
gPVG	1 (Ref.)	0.76 (0.61-0.93)	0.71 (0.57-0.89)	0.56 (0.44-0.73)	0.61 (0.48-0.79)
hPVG	1 (Ref.)	0.78 (0.63-0.97)	0.79 (0.64-0.97)	0.84 (0.66-1.05)	0.68 (0.54-0.87)
uPVG	1 (Ref.)	1.34 (1.08-1.65)	1.30 (1.03-1.65)	1.26 (1.00-1.59)	1.24 (0.98-1.57)

Abbreviations: gPVG, general pro-vegetarian dietary pattern; hPVG, healthful pro-vegetarian dietary pattern; uPVG, unhealthful pro-vegetarian dietary pattern.

Model adjusted for sex (men; women), age (<49; 50-59; 60-69; ≥70 years), social class (low; intermediate; high), tobacco smoking (never; former; current), alcohol consumption (0; 1-2, ≥3 drinks/day) and energy intake (kcal/day).

Supplementary table 14. Sensitivity analysis without missing data for *Helicobacter pilory* infection status: Odds ratios and 95% confidence intervals of gastric cancer according to the adherence to pro-vegetarian dietary patterns (in quintiles, Q) in the StoP Consortium (*n* = 4,151).

	**gPVG dietary pattern**						
	**Q1** (<37)[1]	**Q2** (37-40)	**Q3** (41-43)	**Q4** (44-46)	**Q5** (≥47)	**Per 1 quintile increment**	**Per 5 points increment in adherence**
Controls/cases	696/275	731/195	444/111	809/151	631/108		
Model adjusted	1 (Ref.)	0.80 (0.62-1.01)	0.76 (0.56-1.01)	0.60 (0.46-0.79)	0.62 (0.46-0.84)	0.88 (0.82-0.94)	0.85 (0.79-0.93)
	**hPVG dietary pattern**						
	**Q1** (<49)[1]	**Q2** (49-52)	**Q3** (53-56)	**Q4** (57-60)	**Q5** (≥61)	**Per 1 quintile increment**	**Per 5 points increment in adherence**
Controls/cases	695/208	627/176	742/191	571/138	676/127		
Model adjusted	1 (Ref.)	0.84 (0.65-1.07)	0.81 (0.64-1.04)	0.81 (0.62-1.06)	0.67 (0.51-0.89)	0.92 (0.86-0.98)	0.91 (0.85-0.96)
	**uPVG dietary pattern**						
	**Q1** (<49)[1]	**Q2** (49-53)	**Q3** (54-56)	**Q4** (57-60)	**Q5** (≥61)	**Per 1 quintile increment**	**Per 5 points increment in adherence**
Controls/cases	858/137	743/198	539/149	582/175	589/181		
Model adjusted	1 (Ref.)	1.46 (1.13-1.88)	1.47 (1.12-1.94)	1.43 (1.09-1.88)	1.34 (1.01-1.77)	1.05 (0.99-1.12)	1.06 (1.00-1.13)

Abbreviations: gPVG, general pro–vegetarian dietary pattern; hPVG, healthful pro–vegetarian dietary pattern; uPVG, unhealthful pro–vegetarian dietary pattern.

[1] Adherence range points.

Model adjusted for sex (men; women), age (<49; 50-59; 60-69; ≥70 years), social class (low; intermediate; high), tobacco smoking (never; former; current), alcohol consumption (0; 1-2, ≥3 drinks/day), energy intake (kcal/day) and *Helicobacter pylori* infection (seronegative, seropositive).

Supplementary table 15. Stratified analysis for *Helicobacter pilory* infection status: Odds ratios and 95% confidence intervals of gastric cancer according to the adherence to pro-vegetarian dietary patterns (in quintiles, Q) in the StoP Consortium.

	**gPVG dietary pattern**						
Helicobacter pilory status	**Q1** (<37)[1]	**Q2** (37-40)	**Q3** (41-43)	**Q4** (44-46)	**Q5** (≥47)	**Per 1 quintile increment**	**Per 5 points increment in adherence**
Seropositive (*n*=595)	1 (Ref.)	0.74 (0.41-1.33)	0.74 (0.35-1.58)	0.62 (0.31-1.22)	0.37 (0.14-0.96)	0.83 (0.68-1.00)	0.76 (0.61-0.94)
Seronegative (*n*=3,556)	1 (Ref.)	0.80 (0.61-1.04)	0.76 (0.55-1.05)	0.59 (0.44-0.80)	0.66 (0.48-0.91)	0.89 (0.83-0.96)	0.87 (0.80-0.96)
	**hPVG dietary pattern**						
Helicobacter pilory status	**Q1** (<49)[1]	**Q2** (49-52)	**Q3** (53-56)	**Q4** (57-60)	**Q5** (≥61)	**Per 1 quintile increment**	**Per 5 points increment in adherence**
Seropositive (*n*=595)	1 (Ref.)	0.81 (0.45-1.46)	0.72 (0.40-1.28)	0.44 (0.21-0.92)	0.29 (0.13-0.64)	0.75 (0.63-0.89)	0.77 (0.65-0.91)
Seronegative (*n*=3,556)	1 (Ref.)	0.84 (0.64-1.11)	0.83 (0.63-1.09)	0.89 (0.66-1.19)	0.76 (0.56-1.03)	0.95 (0.89-1.02)	0.93 (0.87-1.00)
	**uPVG dietary pattern**						
Helicobacter pilory status	**Q1** (<49)[1]	**Q2** (49-53)	**Q3** (54-56)	**Q4** (57-60)	**Q5** (≥61)	**Per 1 quintile increment**	**Per 5 points increment in adherence**
Seropositive (*n*=595)	1 (Ref.)	2.95 (1.40-6.19)	2.48 (1.13-5.42)	3.52 (1.67-7.42)	2.01 (0.92-4.37)	1.12 (0.96-1.31)	1.15 (0.98-1.35)
Seronegative (*n*=3,556)	1 (Ref.)	1.33 (1.01-1.75)	1.37 (1.02-1.85)	1.23 (0.91-1.66)	1.30 (0.96-1.75)	1.04 (0.97-1.11)	1.05 (0.98-1.12)

Abbreviations: gPVG, general pro–vegetarian dietary pattern; hPVG, healthful pro–vegetarian dietary pattern; uPVG, unhealthful pro–vegetarian dietary pattern.

[1] Adherence range points.

Model adjusted for sex (men; women), age (<49; 50-59; 60-69; ≥70 years), social class (low; intermediate; high), tobacco smoking (never; former; current), alcohol consumption (0; 1-2, ≥3 drinks/day) and energy intake (kcal/day).

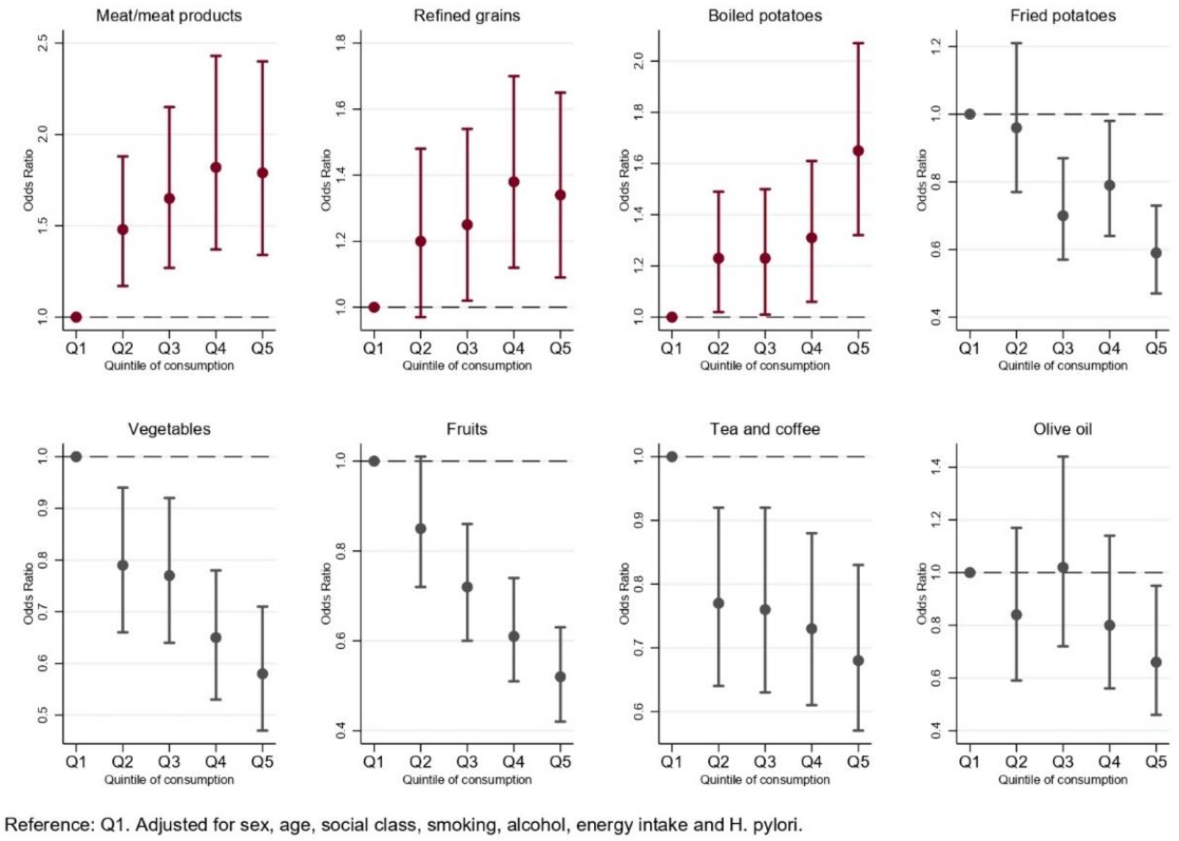

Supplementary Figure 1. Association between selected food groups and gastric cancer risk in the StoP Consortium.
